# The culturable intestinal microbiota of triploid and diploid juvenile Atlantic salmon (*Salmo salar*) - a comparison of composition and drug resistance

**DOI:** 10.1186/1746-6148-7-71

**Published:** 2011-11-17

**Authors:** Leon Cantas, Thomas WK Fraser, Per Gunnar Fjelldal, Ian Mayer, Henning Sørum

**Affiliations:** 1 Department of Food Safety and Infection Biology, Norwegian School of Veterinary Science, NO-0033 Oslo, Norway; 2 Norwegian Food Safety Authority, District Office for Romerike, NO-2381 Brumunddal, Norway; 3 Department of Production Animal Clinical Sciences, Norwegian School of Veterinary Science, NO-0033 Oslo, Norway; 4 Institute of Marine Research (IMR), Matre Research Station, NO-5984 Matredal, Norway

## Abstract

**Background:**

With the increased use of ploidy manipulation in aquaculture and fisheries management this investigation aimed to determine whether triploidy influences culturable intestinal microbiota composition and bacterial drug resistance in Atlantic salmon (*Salmo salar*). The results could provide answers to some of the physiological differences observed between triploid and diploid fish, especially in terms of fish health.

**Results:**

No ploidy effect was observed in the bacterial species isolated, however, triploids were found to contain a significant increase in total gut microbiota levels, with increases in *Pseudomonas *spp., *Pectobacterium carotovorum*, *Psychrobacter *spp., *Bacillus *spp., and *Vibrio *spp., (12, 42, 9, 10, and 11% more bacteria in triploids than diploids, respectively), whereas a decrease in *Carnobacterium *spp., within triploids compared to diploids was close to significant (8% more bacteria in diploids). With the exception of gentamicin, where no bacterial resistance was observed, bacterial isolates originating from triploid hosts displayed increased resistance to antibacterials, three of which were significant (tetracycline, trimethoprim, and sulphonamide).

**Conclusion:**

Results indicate that triploidy influences both the community and drug resistance of culturable intestinal microbiota in juvenile salmon. These results demonstrate differences that are likely to contribute to the health of triploid fish and have important ramifications on the use of antibacterial drugs within aquaculture.

## Background

Artificial triploid fish, that possess three complete sets of chromosomes compared to the more natural state of diploidy that possess two complete chromosome sets, are sterile due to reduced gonadal development and non-functional gametes [1]. Consequently, triploids are appealing to the aquaculture industry as a method for eliminating genetic interactions between wild and cultured stocks and reducing the financial costs associated with early maturation [2,3]. Today, triploids (mainly salmonids) are commercially produced in several countries around the globe including France and Australia [1,4].

Previously, is has been commented that the physiology of triploids is sufficiently different to diploids that they should be treated as a separate species [5]. Of most interest, triploids are composed of cells that are typically 50% larger than diploids due to the accommodation of the extra genetic material within the cell nucleus [6]. However, no size increase of the fish body is achieved through triploidy as the majority of tissues are composed of fewer cells, resulting in similar organ and whole body sizes between the ploidies [6-8]. The reduction in cell number may reduce oxygen delivery [9] and there is also evidence to suggest triploids have a lower optimum metabolic temperature compared to diploids [10], altered nutritional requirements [11], and differences in ontogenetic growth [1]. It is also widely reported that triploids are more susceptible to stress and disease [12-15], although this has yet to be confirmed in controlled experiments [16-19]. In addition, in contrast to male triploids, triploid females typically show no morphological or physiological signs of sexual maturation [8].

Evidence from both mammalian and fish studies suggests the gut microbiota is both influenced by, and has an influence on, host physiology [20-23]. For instance, whilst the gastrointestinal (GI) tract provides an ecological niche for bacterial growth [20], the microbiota present within the GI tract is dependent on host species, strain, ontogenetic growth, stress levels, and gut structure, whereas nutrient processing and absorption, development of the mucosal immune system, angiogenesis, and epithelial renewal are influenced by the gut microbiota [20-25]. Therefore, the gut microbiota plays a significant role in an individual's physiological status. As several of the above listed host factors are either known or suspected to be affected by triploidy (ontogenetic growth, stress response, immunological functioning, and gut structure/cell size and number), it is likely that gut microbiota is also affected by triploidy. Significantly, a changed microbiota may ease the growth and survival of potential fish pathogenic bacteria and also result in a different maturation of the mucosal immunity in young fish.

Previous studies have focused on characterizing the microbiota of different fish species and the biological significance of those bacteria present. For instance, the GI microbiota of several commercially important species has been studied during various life stages, such as in the salmonids [20]. Subsequently, bacteria have been classified as either allochthonous (transient) or autochthonous (permanent), with the allochthonous bacteria supplying the stock from which the autochthonous bacteria may become established. In addition, some bacteria have been identified as pathogens [i.e. *Vibrio*; [26]], involved in enteritis [27], or inhibit the growth of other bacteria [*Carnobacterium *spp.; [28,29]]. These findings have led to attempts to manipulate the gut microflora of fish through pre- and probiotics to improve fish health and growth [30].

A current area of concern within the field of environmental microbiology is the increase in the occurrence of antibiotic resistance among bacteria related to animal production. In this context it is considered that drug resistant bacteria in the environment may potentially transfer their resistance features to not only animal but also human pathogens [31]. This assumption has led to intensified efforts to monitor bacterial resistance, and is extensively done within bacteria of production animals and their environments [[32,33], monitoring schemes, Norway NORM/NORM-VET, Denmark DANMAP, Sweden SVARM]. Significantly, bacteria are capable of transferring antibiotic resistance both within and between species through processes such as conjugation of transferable R-plasmids [34,35]. Traditionally, the use of antibiotics was assumed the main selection factor for increasing antibiotic resistance in aquaculture and the surrounding environment [36,37]; however, recent evidence would suggest that feed and just the presence of an aquaculture facility may also influence the antibiotic resistance levels of bacteria [38,39].

Differences in physiology related to ploidy may influence the gut microbial community and therefore, through a changed microbiota, secondarily affect the growth and health of triploid fish; two key elements in successful aquaculture, however no information is currently available. The objective of this experiment was to compare the culturable intestinal microbiota of cultured triploid and diploid juvenile Atlantic salmon (*Salmo salar*) for differences in the microbial community and the antibacterial resistance profiles of the bacteria based on primary traditional bacterial culturing.

## Methods

The present experiment was approved by the Norwegian Animal Research Authority and performed according to prevailing animal welfare regulations.

### Fish stock and rearing conditions

Fish eggs were provided by Aquagen (Trondheim, Norway) and reared at Matre Research Station, Norway. On Nov 03 2009, eggs (12,000-22,500/female, n = 209,700) from twelve female Atlantic salmon were fertilized by three different males, each male crossed with four different females (male 1 crossed with females 1-4, male 2 with females 5-8 etc), leaving four half-sibling families per male and three groups of independent families. Hydrostatic pressure, which interferes with the release of the second polar body, was used to create triploids [1,40]. Thirty-seven minutes and 30 seconds after fertilization at 8°C, half of the eggs from each female were subjected to a hydrostatic pressure of 655 bar for 6 min and 15 s (TRC-APV, Aqua Pressure Vessel, TRC Hydraulics inc., Dieppe, Canada), giving a total of twenty four groups (12/ploidy). Thereafter, each group was incubated in single incubation trays [Sterner 2003, Sterner Fish Tech AS] in an isolated (UV treatment) flow-through system (water was buffered with seawater to 0.7 ppt salinity; oxygen saturation 95%; pH 6.9) under darkness. The eggs were mechanically agitated to allow dead eggs to be sorted from live eggs at the eye-egg stage on December 21. Eggs from one of the females showed very high mortality at this stage, both as diploid and triploid, and were excluded from the study. After removing the dead eggs (mean 6.7% per female, min 0.9%, max 21%) in each of the remaining twenty two trays, all the family groups within each ploidy were mixed (1 L per female) in a bucket and re-distributed back into six trays (1 L eggs/tray; n = 3 trays/ploidy) leaving three trays/ploidy that each contained a mixture of all three independent families. Hatching started on February 05 2010, and was completed by February 18. On April 26 2010, the yolk sack larvae of each incubation tray were put into single, square, grey, covered, fiberglass tanks (dimensions 1.5 × 1.5 × 0.5 m; water depth 30 cm; water flow 4 L/min/kg fish; n = 3 tanks/ploidy; approx. 5400 larvae/tank) under continuous light (2 × 18 W fluorescent daylight tubes, OSRAM L 18W/840 LUMILUX OSRAM GmbH, Ausburg, Germany) and continuous feeding using automatic disc feeders and a commercial start feed (NUTRA ST 0.5, Skretting AS, Fontaine-les-Vervins, France) that increased in size (up to 1.5 mm pellets, Skretting AS, Norway). On July 22 2010, the fish from each tank were transferred to single, green, fiber glass, 10.7 m^3 ^tanks (Namdal Plast AS, 3 m Ø; water depth 1 m; n = 3 tanks/ploidy; n = 6 tanks). The fish were reared under continuous light (light source as above) from start-feeding onwards. The mean temperature in the period fertilization to start-feeding was 4.9°C (min 3.2°C, max 6.8°C), and in the period start-feeding to August 2010 it was 13.2°C (min 10.7°C, max 15.6°C). The oxygen saturation in the outlet water was always above 80%.

To determine ploidy status, the diameter of the erythrocytes was measured. This method has previously been validated for identification of triploid salmonids, whose erythrocytes are consistently larger than those in diploids [41]. Blood smears were taken from a total of eighty fish per ploidy (n = 160). The diameters of 10 random red blood cells were measured (Image-Pro Plus, version 4.0, Media Cybernetics Silver Spring, MD, USA) on each blood smear. There was no overlap in mean red blood cell diameters between diploid (mean 16.8 μm, max 18.1 μm) and triploid (mean 20.8 μm, min 19.8 μm) individuals, suggesting 100% efficiency of ploidy manipulation.

### Sampling procedure

On Aug 24 2010, twelve juvenile fish (n = 6/ploidy) of unknown sex (mean weight: diploid 25.5 g, triploid 28.0 g; mean length: diploid 112 mm, triploid 118 mm) from six tanks (three tanks/ploidy; two fish/tank) were euthanized by a sharp blow to the head followed by decapitation and sampled for gut microbiology. Sterile latex gloves or latex gloves wiped with 70% ethanol were used throughout the dissection. The underside of the fish was sterilized using 70% ethanol and the ventral belly surface was opened with a sterile surgical blade and forceps. The spleen, gall bladder, liver, and fat deposits surrounding the GI tract were gently removed to expose the peritoneal cavity without disturbing the intestine. The intestine was divided into foregut, midgut, and hindgut as previously described [42]. Thereafter, the intestinal content from within each gut section was squeezed into separate sterile tubes and the appropriate 10-fold dilutions were prepared in physiological saline. Then 100 μl aliquots were pipetted onto the surface of 5% cattle blood agar (blood agar base no 2, Difco) and Brocalin agar (Merck, Darmstadt, Germany) both with 1% NaCl. Dry feed samples and water samples were also appropriately diluted and streaked onto blood agar and Brocalin agar. The plates were incubated at 12°C (± 1) and inspected regularly for up to 4 weeks.

### Bacterial isolation, genus identification, and quantification

After incubation, each morphologically distinct colony (form, size, surface, color, texture, elevation, margin, and hemolysis) was coded. To obtain pure growth, two randomly selected representatives of all the coded bacterial colonies from each section of the intestine, were sub-cultured on blood agar. Afterwards, pure cultured colonies were checked for motility under a light microscope (Leitz, 301-314.001, Germany) at 1000 × magnification using a glass slide with suspended and Gram stained exponentially grown bacteria in physiological salt water [43]. Catalase and oxidase activity were tested as well as the ability to reduce nitrate. The IMViC reactions; indol production, methyl red test (fermentation of glucose), Voges-Proskauer reaction (diacetyl-production) and the ability to use citrate as the sole carbon source (Simmons citrate medium) were tested. H_2_S production, gelatinase, urease and β-galactosidase (ONPG) production and ability to degrade glucose, trehalose, lactose, maltose, mannitol and esculin were tested [Table 1].

**Table 1:** Phenotypical properties of bacterial isolates.

Gram stain and Colony descriptions	Haemolysis	Motility	Catalase	Oxidase	Nitrate reductase	Indole	Methyl-Red	Voges-Proskauer	Citrate Utilization	H_2_S Production	Gelatinase	Urea hydrolysis	O/129 disk	ONPG	Glucose	Trehalose	Lactose	Maltose	Mannitol	Esculin	Organisms and No of strains
GN rods, short, plump, in coccoid pairs or chains of variable length (Translucent to opaque, convex, entire colonies 1.5-2 mm)	-	-	+	-	-	-	-	-	V	-	-	-	-	-	V	-	V	-	-	-	*Acinetobacter *spp. n: 24
GN slightly curved rods, (Large, smooth, yellowish-white mucoid 2-4 mm colonies)	V	V	+	+	V	-	-	-	+	-	V	V	-	V	-	-	-	V	V	-	*Pseudomonas *spp. n: 72
GN rods (Flat, greenish, 1-2 mm colonies)	V	-	+	+	V	-	-	-	+	-	+	V	-	-	-	V	-	V	V	-	*P. fluorescens *n: 72
GN coccobacilli (Slightly, convex, cream colored 2 mm colonies)	-	-	-	+	V	-	-	-	-	-	-	V	-	-	-	-	-	-	-	-	*Psychrobacter *spp. n: 48
GN rods (Opaque, circular, convex, white to pink entire colonies 2-3 mm)	-	+	-	-	V	-	V	+	+	-	+	-	-	+	+	+	-	+	+	V	*Serratia *spp. n: 24
GN curved or comma shaped rod (smooth, moist, opaque-white\yellow green, round 2-3 mm colonies)	V	+	+	+	+	+	V	-	V	-	V	V	+	V	+	+	V	V	V	V	*Vibrio *spp. n; 72
GN regular rods (Shiny, orange-yellow, circular, smooth, concave, 2 mm colonies)	-	-	+	-	+	ND	ND	ND	+	ND	+	+	ND	ND	+	+	+	ND	ND	-	*Pectobacterium carotovorum *n; 48
GP irregular rods with jointed rods 'V-shape' (Yellow-Greenish-metallic center,1-2 mm colonies)	-	-	-	+	V	-	-	-	-	-	V	V	-	-	-	V	V	V	V	-	*Arthrobacter *spp. n; 48
GP straight long-narrow rods in chain, contain oval endospore (Gray-white, dry appearance 3-4 mm)	V	+	+	V	V	V	V	V	V	-	V	V	V	V	+	+	V	V	V	V	*Bacillus *spp. n; 48
GP slightly slender singly rods (Whitish, round, convex, 1-2 mm colonies)	-	-	-	-	-	-	-	+	+	-	-	-	-	-	V	+	+	V	+	+	*Carnobacterium *spp. n; 48
GP cocci singly, in pairs (Circular, convex to slightly peaked, smooth, glossy, opaque 1-5 mm colonies)	-	ND	+	-	-	ND	ND	ND	ND	ND	ND	+	ND	-	+	+	V	+	V	+	*Staphyloccocus *spp. n: 48
Negative Control (Sterilized 0.9% NaCl)	-	-	-	-	-	-	-	-	-	-		-	-	-	-	-	-	-	-	-	*-*

**Key: **GP = Gram Positive; GN = Gram Negative; ONPG: *ortho *-nitrophenyl-, 3-D-galactopyranoside. ND = Not done; + = 90% or more strains are positive; - = 90% or more strains are negative; V = Isolate variability (% 11.3-87.6).

From both fish lines a total of 552 different isolates were identified to the genus level based on morphological and biochemical characteristics and compared with Bergey's Manual of Determinative Bacteriology, Ninth Edition (2000). The total count of named viable bacteria was indicated in terms of colony forming units per mg (cfu/mg).

### Antibiotic susceptibility testing of isolated strains

Two identified colonies of *Acinetobacter *spp., *Pseudomonas *spp., *Pseudomonas fluorescens*, *Serratia *spp., *Vibrio *spp. and *Psychrobacter *spp. were randomly selected from each individual fish. All coded bacterial colonies were identified from the hindgut, with the exception of *Acinetobacter *spp. that was only sampled from the foregut. Antimicrobial susceptibility of a total of 144 isolates was tested against tetracycline (Tet 80 μg), sulphonamide (Sul 240 μg), trimethoprim (Trim 5.2 μg), streptomycin (Str 100 μg), gentamicin (Gt 240 μg), nalidixic acid (Nal 130 μg) and chloramphenicol (Chl 60 μg) by the disc diffusion method [Neo-Sensitabs, Rosco, Taastrup, Denmark]. Inhibition zones were measured and resistance categorization was carried out according to Minimal Inhibitory Concentration (MIC) break points of the Norwegian AFA group (2005, 2006), described in 'User's guide Neo-Sensitabs^®^' [http://www.rosco.dk]. Intermediate zones were recorded as resistant.

### DNA isolation, 16S rDNA amplification

DNA extraction was performed from a loop-full of overnight growth, identified from 18 multidrug resistant (≥ 3 drugs), Gram negative isolates, including two representatives from a biochemically unclassified Gram negative bacteria group, using the DNAeasy Blood & Tissue Kit^® ^[Qiagen S.A., France], according to the manufacturers guidelines. Following nucleic acid purification, the 5' part of the 16S rRNA gene (corresponding to *Escherichia coli *positions 10 to 806) was amplified using primers V1 [5'-AGA GTT TGA TCA TGG CTC AGA] and V3 [5'-GGT TAC CTT GTT ACG ACT TC]. Briefly, cycling parameters included an initial denaturizing step for 3 min at 94°C; 30 cycles of 30 s at 94°C, 30 s at 56°C, and 2 min at 72°C; and a final extension for 10 min at 72°C. Two μl of the DNA extract was used for amplification in a total volume of 25 μl containing 2.5 μl 10 × PCR Buffer, 1 μl 50 mM MgCl_2_, 1 μl 10 mM dNTP mix, 5 pMol each of forward and reverse primers, 1 U of Taq DNA polymerase [Fermentas, Vilnius, Lithuania] and 16.4 μl sterile nuclease-free PCR grade water. DNA amplifications were performed in a GeneAmp 9700 PCR system thermocycler [Applied Biosystems, Foster City, USA]. Positive (*Escherichia coli *NVH 1067/03) and negative control (sterilized dH_2_O) samples were included in all amplifications. The PCR products were analyzed on 1.5% agarose gel stained with SYBR Safe^® ^DNA Gel Stain [Invitrogen]. After electrophoresis at 100 V for 60 min, DNA bands were visualized by a Gel Doc™ XR+ Imaging System [Bio-Rad, Hercules, CA].

### Sequence analysis

Amplicons were purified by using QIAquick PCR Purification Kit^® ^[Qiagen S.A., France] and sequenced by GATC laboratories [Konstanz, Germany] with V1 and V3 primers. Afterwards 16S rRNA gene sequences were compared with those available in the GenBank, EMBL, and DDBJ databases using a two-step procedure. A first search was performed with the FASTA algorithm of the Wisconsin GCG program package [44]. All positions showing differences to the best-scoring reference sequence were visually inspected in the electropherogram, and the sequence was corrected manually if necessary as previously described [45]. Thereafter, a second search was done using BLASTN. Undetermined nucleotides (designated N) in either the determined sequence or the reference sequence were counted as matches. All derived sequences have been submitted to GenBank, with accession numbers listed in [Table 2].

**Table 2:** The GenBank accession numbers of partial 16s rRNA gene sequences of intestinal MDR Gram negative triploid and diploid Atlantic salmon isolates.

Fish	Source	Drug Resistance	Strain and Gene	GenBank accession number
Diploid	Foregut	Chl, Nal, Tet	*Acinetobacter *sp. Cantas1,16S ribosomal RNA gene, partial sequence	JN609530
Diploid	Foregut	Nal, Str, Tet, Trm	*Acinetobacter *sp. Cantas2 16S ribosomal RNA gene, partial sequence	JN609531
Diploid	Foregut	Nal, Tet, Trm	*Acinetobacter *sp. Cantas3 16S ribosomal RNA gene, partial sequence	JN609532
Triploid	Foregut	Nal, Str, Tet. Trm	*Acinetobacter *sp. Cantas4 16S ribosomal RNA gene, partial sequence	JN609533
Triploid	Foregut	Str, Sul, Tet	*Acinetobacter *sp. Cantas5 16S ribosomal RNA gene, partial sequence	JN609534
Triploid	Foregut	Nal, Sul, Tet	*Acinetobacter *sp. Cantas616S ribosomal RNA gene, partial sequence	JN609535
Triploid	Foregut	Str, Tet. Trm	*Acinetobacter *sp. Cantas7 16S ribosomal RNA gene, partial sequence	JN609536
Diploid	Hindgut	Sul, Tet, Trm	*Pseudomonas *sp. Cantas8 16S ribosomal RNA gene, partial sequence	JN609537
Diploid	Hindgut	Nal, Sul, Tet	*Pseudomonas *sp. Cantas9 16S ribosomal RNA gene, partial sequence	JN609538
Triploid	Hindgut	Sul, Tet, Trm	*Pseudomonas sp*. Cantas10 16S ribosomal RNA gene, partial sequence	JN609539
Triploid	Hindgut	Sul. Tet, Trm	*Pseudomonas *sp. Cantas11 16S ribosomal RNA gene, partial sequence	JN609540
Triploid	Hindgut	Nal, Sul Tet, Trm	*Pseudomonas *sp. Cantas12 16S ribosomal RNA gene, partial sequence	JN609541
Triploid	Hindgut	Nal, Tet, Trm	*Pseudomonas *sp. Cantas13 16S ribosomal RNA gene, partial strain	JN609542
Diploid	Hindgut	Sul, Tet, Tm	*Pseudomonas fluorescens *Cantas14 16S ribosomal RNA gene, partial sequence	JN609543
Triploid	Hindgut	Nal, Tet, Sul	*Pseudomonas fluorescens *Cantas15 16S ribosomal RNA gene, partial sequence	JN609544
Diploid	Hindgut	Tet, Str, Sul	*Psychrobacter *sp. Cantas16 16S ribosomal RNA gene, partial sequence	JN609545
Triploid	Hindgut	Tet, Trm, Sul	*Psychrobacter *sp. Cantas17 16S ribosomal RNA gene, partial sequence	JN609546
Triploid	Hindgut	Chl, Str, Tet, Trim	*Serratia *sp. Cantas18 16S ribosomal RNA gene, partial sequence	JN609547

### Statistical analysis

#### GI microflora

The data was recorded in a Microsoft Office Excel 2003^® ^spread sheet and then transferred to JMP^® ^8.0 Statistical Discovery Software from SAS for statistical analysis. After descriptive analyses of data using standard summary measures and graphical representation, two least-squares models were established, with total bacterial count and individual bacterial species count as the outcomes. First, the effect of ploidy (ordinal, diploid versus triploid) and gut section (ordinal, foregut, midgut and hindgut) on total bacteria count was tested. Secondly, a similar model was established for individual bacterial species. The model gave a standard analysis of variance table and the corresponding regression coefficients. Model fit was assessed using graphical techniques, plotting actual versus predicted values, and residuals were assessed using the normal quantile plot. To investigate for possible tank effects, parametric and non-parametric tests were used with i) the tank considered the unit of measure (n = 3/ploidy) and ii) the tank included within a nested model design (with individual fish the unit of measure, n = 6/ploidy). No tank effects were observed, with the results (the effect of ploidy on gut microbiota) remaining the same whether tank was included in the model or not (data not shown). Graphs were made in Microsoft Word Excel and JMP^®^. Results were considered significant at *P *< 0.05.

### Antibacterial resistance

Antibiotic sensitivity records for each isolate at ploidy level were coded in a Microsoft Excel 2003^® ^spread sheet and the mean antibiotic resistance displayed as a histogram. For further statistical analysis, all data were transferred to Stata [Stata SE/10 for Windows, Stata Corp., College Station, TX]. For each antibiotic, an ordered linear logistic regression model analysis was built and odds ratio and 95% confidence intervals detected.

## Results

Fish growth was not affected by the triploid treatment (data not shown), and all fish appeared healthy with no gross deformities or lesions at the macroscopic level within the gut after visual inspection. Furthermore, by naked eye, there was no discernable difference in the amount of digesta within the gut of individual fish.

### Fish rearing environment

Bacterial counts in the food source included 3.7 × 10^4 ^cfu/g *Staphylococcus *spp., 5.5 × 10^3 ^cfu/g *Bacillus *spp., 1.1 × 10^3 ^cfu/g *Pseudomonas *spp., and 4.8 × 10^2 ^cfu/g *Carnobacterium *spp. Bacterial counts from the system inflow water contained 8.0 × 10^5 ^cfu/ml *Pseudomonas *spp., 2.0 × 10^3 ^cfu/ml *P. fluorescens*, and 1.9 × 10^2 ^cfu/ml *Psychrobacter *spp. In the tank water, the culturable bacteria consisted of a mixture of intestinal and feed bacterial flora, where *Bacillus *spp. and *Vibrio *spp. dominated, with differences between the tanks for each ploidy, triploids having 10^2 ^- 10^3 ^cfu/ml more *Bacillus *spp. and *Vibrio *spp. respectively, than diploids. For the other bacteria present, *Acinetobacter *spp., *Carnobacterium *spp., *Psychrobacter *spp., *Pectobacterium carotovorum*, *Arthrobacte*r spp., and *Pseudomonas *spp. ranging from 10^3 ^- 10^5 ^cfu/ml, there was no difference between the tanks of each ploidy.

### GI microflora

**Figure 1:**
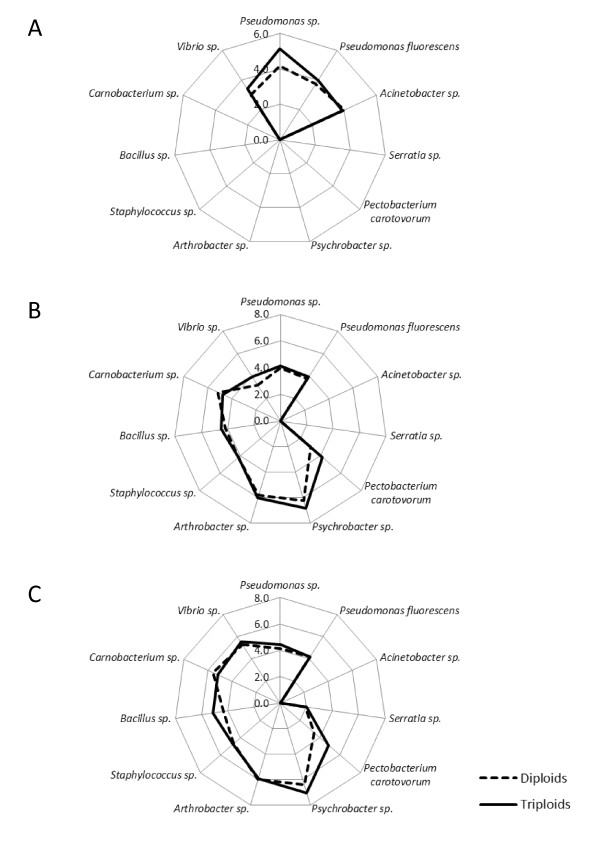
**Spider graphs showing the mean bacterial levels (CFU/mg) within (A) foregut, (B) midgut, and (C) hindgut of juvenile triploid and diploid Atlantic salmon**.

**Figure 2:**
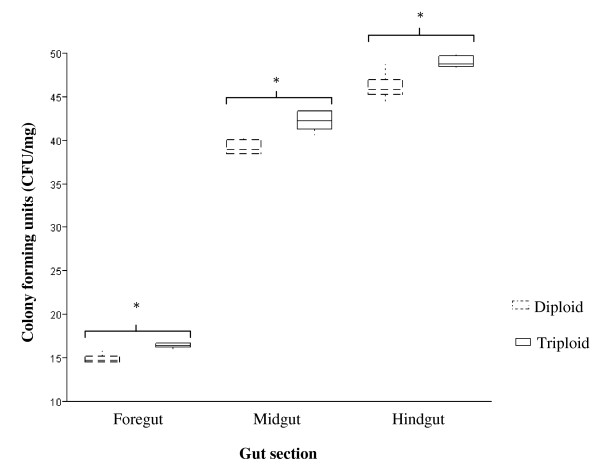
**Box plot showing the total bacteria counts within the different gut sections of triploid and diploid juvenile Atlantic salmon**. There was a significant difference (*p *< 0.05) in colony forming units (CFU) between all gut sections. Compared to diploids, triploid salmon had a statistically significantly (*p *< 0.05, indicated by an asterisk) greater number of bacteria within each gut section.

On the basis of physiological and biochemical identification 10 different bacterial genera were isolated and characterized within the GI tract of each ploidy and these corresponded to the genetic analysis [Tables 1 and 2]. There was no effect of ploidy on the bacterial diversity of the GI tract, or from which gut section the individual species were isolated [Figure 1]. No bacteria dominated the bacterial counts either as a percentage of the whole gut, or within specific gut sections, with *Pseudomonas *spp., *P. fluorescens*, *Pectobacterium carotovorum*, *Psychrobacter *spp., *Arthrobacter *spp., *Staphylococcus *spp., *Bacillus *spp., and *Carnobacterium *spp., each being between 8 and 13% of the total GI bacterial counts. There was a statistical significance in level of total bacterial counts from the foregut to the hindgut in both ploidy; however triploids had significantly greater total bacterial counts within each gut section compared to diploids [Figure 2]. Triploids had on average 7.3% more bacteria within the whole gut than diploids (max diploid and min triploid value 102.1 and 105.8 cfu/ml respectively), with the largest increase observed within the foregut (10%).

Triploidy had a significant effect on a number of the individual bacterial species isolated [Table 3]. Significantly increased levels of *Pseudomonas *sp., *Pectobacterium carotovorum*, *Psychrobacter *spp., *Bacillus *spp., and *Vibrio *spp., were observed in triploid fish compared to diploids (triploids having on average 12, 42, 9, 10, and 11% more bacteria than diploids, respectively), whereas a decrease in *Carnobacterium *spp., within triploids compared to diploids was close to significant (*P *= 0.1, diploids having on average 8% more bacteria than triploids). Where significant increases in bacteria were observed between the ploidies, the increase was not always universal along the whole gut. The increase in *Pseudomonas *spp., and *Vibrio *spp., over diploid counterparts was more pronounced in the forgut and midgut respectively, compared to *Pectobacterium carotovorum*, *Psychrobacter *spp., and *Bacillus *spp., where there was a similar increase in triploid bacterial populations along the entire gut length [Figure 1].

**Table 3:** *P *values for the effect of ploidy and gut section on bacterial counts within the gastrointestinal tract of juvenile Atlantic salmon.

Bacteria	Gut section	Ploidy
*Pseudomonas *spp.	0.0043	0.0013
*Pseudomonas fluorescens*	0.0246	0.1522
*Acinetobacter *spp.	0.0001	0.5552
*Serratia *spp.	0.0001	0.6727
*Pectobacterium carotovorum*	0.0001	0.0001
*Psychrobacter *spp.	0.0001	0.0150
*Arthrobacter *spp.	0.0001	0.4881
*Staphylococcus *spp.	0.0001	0.4250
*Bacillus *spp.	0.0001	0.0005
*Carnobacterium *spp.	0.0001	0.0989
*Vibrio *spp.	0.0001	0.0001

### Antibacterial resistance

No ploidy effect was observed on bacterial multiple drug resistance, or the numbers of antibiotics the bacteria were resistant to. For diploid and triploid isolated bacteria respectively, the frequency of resistant bacteria to two antibacterials was 16 and 20.8% with 9.7 and 13.2% of those resistant to more than two antibacterials. All strains were susceptible to gentamicin, whilst the least resistance was demonstrated against chloramphenicol which inhibited more than 95% of the strains.

**Figure 3:**
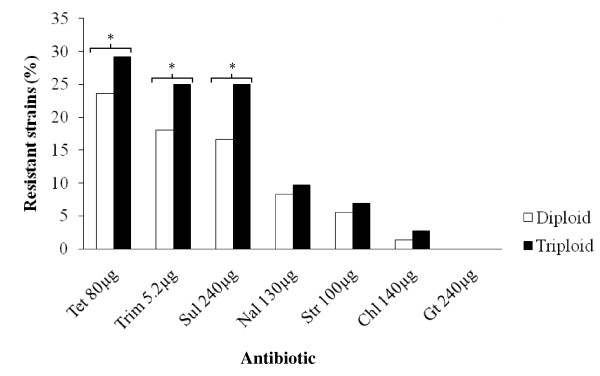
**The average frequency distributions of antibiotic resistant gram negative isolates (N = 144) among triploid and diploid fish**. There was a trend of increased antibacterial resistance from bacteria isolated from triploids compared to those bacteria isolated from diploids, this was statistically significant (*p *< 0.05, indicated by an asterisk) for some antibiotics.

For all the antibiotics where resistance was observed, there was a general trend of increased resistance in isolates from triploid fish compared to isolates from diploid fish [Figure 3]. Statistically, the presence of tetracycline (OR = 1.117; 95% CI = 0.52-2.37), trimethoprim (OR = 1.609; 95% CI = 0.71-3.64) and sulphonamide (OR = 1.46; 95% CI = 0.65-3.26) resistance was significantly higher in triploid fish intestinal Gram negative isolates compared to their diploid counterparts, whereas no significant differences related to ploidy were observed for streptomycin (OR = 0.496; 95% CI = 0.38-7.25), nalidixic acid (OR = 0.97; 95% CI = 0.29-3.16), or chloramphenicol (OR = 0.97; 95% CI = 0.23-7.09).

## Discussion

To our knowledge, this is the first study into the intestinal microbiota of triploid Atlantic salmon, and the first into any species of triploid fish. The objective was to compare the GI microflora and the antibiotic resistance profiles of the intestinal microflora of Atlantic salmon after chromosome set manipulation (triploidy) to see if it resulted in an altered physiological profile. No differences were observed in the diversity of bacteria isolated from the gut of triploid or diploid fish, however, triploid fish were found to contain greater total bacterial counts within each gut section [Figure 2], although this was not due to a general increase in all the isolated bacteria [Table 3]. For specific bacteria, triploids were found to contain significantly more *Pseudomonas *sp., *Pectobacterium carotovorum*, *Psychrobacter *spp., *Bacillus *spp., and *Vibrio *spp., whereas *Carnobacterium *spp. was close to being significantly lower in triploids compared to diploids. These results suggest the physiological differences associated with triploidy have an influence on the gut microbiota.

Bacterial culturing and 16S rRNA PCR were used in this study to identify and quantify the bacteria within the fish gut. It is acknowledged that traditional culturing may only identify 11-50% of the present bacteria [46,47]. Additionally, competitive differences between the bacteria on the culture medium compared to the fish gut may lead to misrepresentation of the microbiota community [42], and the media used in this study, blood agar and Brocalin agar, are not traditionally used in fish studies and may not have supported growth of *Lactobacillus *spp., previously found to be dominant in the GI tract of Atlantic salmon [48]. Metagenomic analysis may have led to a more realistic representation of the GI microflora in this study, however, this method is relatively underdeveloped in Atlantic salmon and not comparable to the existing literature that uses methods that are still routinely, and historically, used in the field of microbiology. As such, previously identified increases in the level of bacteria from the foregut to the hindgut were observed in this study [23,42], along with the appearance of certain bacteria inhabiting the different gut sections within salmonids [20,42,49]. Therefore, the authors believe the results of this study are reliable for making an accurate comparison related to ploidy within this study.

Differences were observed in the culturable bacteria levels between the triploid and diploid tank water. Increases in *Bacillus *spp. and *Vibrio *spp. within triploid tanks were consistent compared to diploid tanks, however the levels of the other isolated bacteria were similar. All tanks were supplied with the same inflow water that contained neither *Vibrio *spp., nor *Bacillus *spp., however, feed did contain *Bacillus *spp. Therefore, increases in *Bacillus *spp. could be explained by an increase in uneaten feed in the triploid tanks; however, feed intake was not assessed and uneaten feed was removed by the self-cleaning design of the tank, and this would not explain the increase in *Vibrio *spp. Alternatively, the increases in *Vibrio *spp. and *Bacillus *spp., could be explained by the release of allochthonous bacteria that are only transient in the gut and are released over time in faeces. Therefore the increase in *Bacillus *spp., and *Vibrio *spp., in triploid compared to diploid tank water could be expected due to the increases in these two bacteria observed within the triploid gut. However, this was not the case for *Pseudomonas *spp., *Pectobacterium carotovorum*, and *Psychrobacter *spp. that were found in equal quantities in tank water from both ploidies, despite being found to have increased numbers within the triploid gut. The reasons for this are unknown, but may be related to the ability of different bacterial species to adhere to the intestinal mucosa as part of the autochthonous flora and therefore not be released in such great quantities in the faeces, or the ability of these bacteria to survive and compete within the tank water.

Limitations in our study include no control for the effect of pressure treatment independent of ploidy, and the effect of genetic variation within the study fish. Firstly, the current evidence would suggest that triploid induction procedures (when optimized) do not influence triploid performance [12,13,16], although Malison et al. [50] concluded otherwise, this study failed to have an adequate control. With regards to the genetic composition of the study population, it is recognized family effects are observed on triploid survival and physiological performance [51] and this may influence gut microbiota, however we did not test for this. Therefore, the study should be repeated with an emphasis on the family effect on gut microbiota in fish of both ploidy.

Previously, 10 of the 11 isolated bacteria have been recorded in Atlantic salmon [27,42,52,53]. In addition, the levels of bacteria isolated in this study agree with those previously found in Atlantic salmon [42]. However, we could not find any previous report of *Pectobacterium carotovorum *subsp. *carotovorum*, previously isolated from Chinese cabbage [Zhang et al. 2008, unpublished data], in Atlantic salmon. Its source and biological significance in this study are unknown.

Our results suggest the triploid gut provides a different environment to that of the diploid gut, based on bacterial levels. Unfortunately, no literature exists on the triploid gut, or triploid physiology that may affect bacterial growth, therefore some of the major factors that influence gut microbiota cannot be discussed (i.e. gut mucous, physiochemical environment) [52,54,55]. Although no significant differences on weight, length, or condition factor were seen within this study, significant differences were seen in the growth (triploids were heavier and longer), condition factors (triploids had a lower value), and the hepatosomatic index (higher in triploids) in pre-smolts. Previously, differences in gut microbiota have been identified between fast and slow growing members of fish from the same population [25], and may explain our results. Alternatively, we also observed differences in the leucocyte composition (neutrophilic and B-cells proportions) in the same study fish three weeks after seawater transfer (Fraser et al, in preparation). It is well established that the gut microbiota influences the development of the immune system in mammals [56] and evidence suggests the same occurs in fish [22]. Therefore, it is possible that differences in the immune system related to ploidy (i.e. leucocyte proportions) are influencing the size and composition of the gut microbiota through interactions with mucosal immune cells, or the mechanisms by which the microbiota influences the immune development in fish may be altered by the ploidy status. Cortisol levels and female sex hormone levels have been found to be lower in triploids compared to diploids [18,57,58], and these may also influence the immune system in fish [59-65]. However, the majority of studies have observed no differences in cortisol [18,62-65] due to ploidy and, up until the time of maturing sex hormone levels are typically below detectable levels in salmon and therefore unlikely to influence the immune system in this study. A final explanation is that of differences in cell size and number relating to ploidy. It is well established that cell size increases with increasing numbers of chromosome sets [6]. This phenomenon is more apparent in cells where the nucleus occupies a greater proportion of the total cell volume, so may have little effect on some intestinal cells (i.e. enterocytes). However, triploids have organs of equal sizes to diploids due to reductions in cell number [6,7], and it may be that triploids have fewer cells along the gastrointestinal tract, such as goblet cells, altering the conditions within the gut. Therefore, differences in the ontogenetic growth, the immune system, or gut environment in the triploids may explain our results.

A difference in the gut microbiota between the ploidies could have a number of implications on triploid aquaculture practices. For instance, *Vibrio *spp., are known to be opportunistic pathogens and causative agents of disease and mass mortalities [26], *Pseudomonas *spp., *Bacillus *spp, and *Carnobacterium *spp., are capable of inhibiting pathogenic bacteria and improving survival *in vivo *after challenge tests [47,66], *Psychrobacter *spp., and *Arthrobacter agillis *have been linked to enteritis in Atlantic salmon [27], and *Bacillus *spp., and *Carnobacterium *spp., are commonly used in fish pro- and pre-biotics [23,30]. These results suggest that the susceptibility of triploids to opportunistic pathogens (important in both fish and human health) or potential probiotic treatments may be increased in comparison to diploids. Indeed, several studies have reported triploids to be more prone to disease than diploids [12-15].

Despite no known antibiotic exposure within our study population, we detected a variety of drug resistance patterns within bacterial isolates. Previous studies have reported antibacterial drug resistance in various aquatic environments [67-69] at levels higher than those found within our study, possibly as a result of antibiotic use that exerts an ecological pressure on bacteria [70]. However, increased levels of antibiotic resistant bacteria, over that of the local environment, have been reported in aquaculture facilities that are not utilizing antibiotics [39], and the reasons for this remain unclear.

Triploids were found to have consistently higher levels of antibacterial resistance in their bacterial isolates than in diploids; with 3 of the 6 effective antibacterials significantly affected by ploidy [Figure 3] but no ploidy effect was seen on the occurrence of multiple drug resistance. We suggest two possible explanations for the first observation i) possible physiological differences between the two ploidies (as discussed above) impacting upon the host-bacterium interaction, and ii) the significantly greater levels of bacteria found within the triploid compared to diploid intestine might provide better conditions for more efficient bacterial colonization, which is very critical for horizontal gene transfer [71]. Unfortunately, both of these hypotheses were outside the scope of this study.

## Conclusions

This study would suggest the altered physiology of triploid fish affects the numbers of microbiota within GI tract and their drug resistance profiles. The observation of altered drug resistance profiles between triploid and diploid fish would also make for an interesting follow up study to determine the factors that cause such a phenomenon. It would also be of interest to see how the observed differences in bacterial communities are affected by fish age, genotype, and rearing environment, as these factors are known to alter the microbiota of the GI tract in diploids. It has also been observed that triploids can demonstrate altered growth patterns compared to diploids, such as delayed time of first feeding and slower growth in the juvenile phase, and the effects on/of the GI microbiota may be of interest.

## Authors' contributions

LC contributed to the establishment of the collaborations, design, performed the sampling and all traditional bacterial culturing, molecular genetic studies, statistical analysis, data collection, drafting and writing of the manuscript. TF contributed to the establishment of the collaborations, design, data collection, data analysis, and drafting and writing the manuscript. P-GF contributed to funding, design, acquisition of the fish, and writing the manuscript. IM contributed to acquisition of funds, the supervision, and drafting of the manuscript. HS contributed to acquisition of funds, the supervision, and drafting and writing the manuscript. All authors read and approved the final manuscript.
